# Prognostic value of the c-reactive protein/prognostic nutritional index ratio after hip fracture surgery in the elderly population

**DOI:** 10.18632/oncotarget.18135

**Published:** 2017-05-24

**Authors:** Hanru Ren, Lianghao Wu, Wankun Hu, Xiuzhang Ye, Baoqing Yu

**Affiliations:** ^1^ Department of Orthopaedics, Shanghai Pudong Hospital, Fudan University, Pudong Medical Center, Shanghai, China

**Keywords:** C-reactive protein/prognostic nutritional index, mortality, hip fracture, elderly population

## Abstract

**Background:**

More and more older patients receive the surgery after hip fracture. However, the mortality rate is high. Prognostic nutritional index (PNI) is associated with prognosis in hip fracture patients. In the current study, we proposed a novel prognostic score, named c-reactive protein/PNI ratio (CRP/PNI ratio), for predicting the prognosis for geriatric orthopedic population.

**Methods:**

This is a prospective study. Eighty cases of hip fracture surgery in the elderly population were studied to reveal the relationship between the CRP/PNI ratio and the clinicopathological characteristics of the elderly patients. Clinical data included age, sex, weight, length of stay, duration of surgery, comorbidity, and biological data were collected. The primary endpoint was the 1-year mortality rate.

**Results:**

Cox regression and log-rank tests were used to evaluate the correlation of CRP/PNI to the one-year mortality. The one-year mortality rate was low in the patients with a low CRP/PNI ratio (*P* < 0.001). Univariate and multivariate survival analyses proved that CRP/PNI was an important factor to predict the one-year mortality rate of the geriatric hip fracture surgery patients.

**Conclusion:**

Low CRP/PNI ratio was significantly associated with low one-year mortality rate in older patients after hip fracture surgery.

## INTRODUCTION

Hip fracture is a fracture of the upper part of the femur, and has high incidence in the elderly [1]. Hip fractures in aged people leads to high mortality, high risk of postoperative complication, and impaired quality of life [2–5]. The mortality is increased from 8.4% to 36% in the first postoperative year [6].

The risk factors for mortality following hip fracture surgery was demonstrated in some studies [7], we hope to reveal the clinically relevant biomarkers to evaluate prognosis and complication events. It has been reported that serum albumin level and lymphocyte count served as an independent prognostic factor in hip fracture patients [8]. The prognostic nutritional index (PNI) is calculated by the serum albumin concentration and the peripheral blood lymphocyte count [9]. Albumin is an indicator of nutritional status which is correlated with post-operative complications [10]. The PNI can be used to assess the immunological and nutritional status of surgery patients [11], and estimated according to the following formula: 10 × serum albumin (g/dL) + 0.05 total lymphocyte count (/mm^3^) [12]. C-reactive protein (CRP) is an inflammatory marker which is closely related to the infections and outcomes in orthopaedics [13]. And PNI is associated with cancer mortality and has a prognostic value [14].

The correlation of CRP/PNI ratio and mortality after hip fracture surgery, especially in elderly population remains unknown. This study is aim to evaluate the predictive value of CRP/PNI ratio in elderly patients after orthopedic surgery, and compare it with clinical variables.

## RESULTS

**Figure 1:**
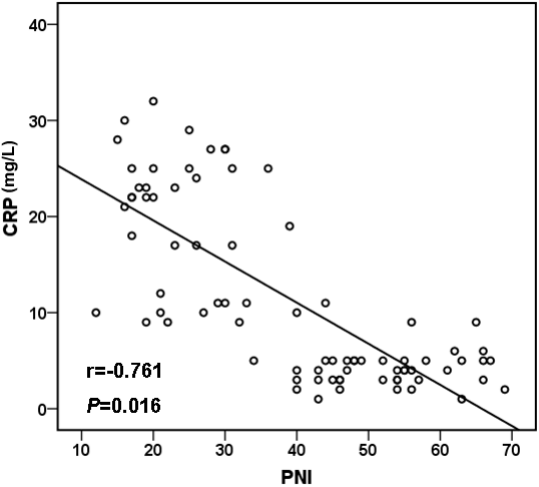
Pearson correlation A signifcant negative correlation between CRP and PNI (r=-0.761, *P* =0.016). CRP: C-reactive protein; PNI: prognostic nutritional index.

We estimated the CRP/PNI ratio in 80 patients, 35 (43.75%) of whom were men and 45 (56.25%) were women. The results are presented in Figure 1 and summarized in Table 1; the mean CRP and PNI were 11.3 ± 9.6 mg/L and 41.3 ± 11.0, respectively. An inverse correlation was found between CRP and PNI with a correlation coefficient of -0.761 which was shown in Figure 1 (*P* =0.016). The relationships between the CRP/PNI ratio and clinical characteristics were shown in Table 1. Based on the receiver operating characteristics (ROC) analyses, the best cut-off value of CRP/PNI ratio was 0.10. Then, patients were divided into two groups: patients with CRP/PNI ratio ≤0.10 and patients with CRP/PNI ratio >0.10. There were 42 (52.5%) patients with CRP/PNI ratio ≤0.10 and 38 (47.5%) patients with CRP/PNI ratio >0.10. CRP/PNI ratio is correlated significantly with CRP (*P* <0.001), leukocytosis (*P* =0.003), lymphocyte counts (*P* <0.001), hypoalbuminemia (*P* =0.001), PNI (*P* <0.001) and survival (*P* <0.001), but no relationships were found between CRP/PNI ratio and other factors such as comorbidity, platelet count and anemia.

**Table 1:** The CRP/PNI ratio and clinicopathological parameters

	Case(n)	CRP/PNI ratio		*p* Value
		≤ 0.10	> 0.10	
Age (years)	86±5	85±7	87±3	0.192
Men	35	18	17	0.301
Weight (kg)	62 (49–78)	61 (45–74)	63 (55–78)	0.762
Length of stay (day)	7 (5–14)	6 (5–10)	9 (6–14)	0.162
Duration of surgery (min)	140 (111–170)	140 (111–170)	140 (111–170)	0.912
Comorbidity				
Diabetes mellitus	34	17	17	0.684
Cardiovascular disease	24	13	11	0.726
Hypertension	63	32	31	0.710
Renal disefficiency	21	12	9	0.691
CRP, mg/L				
<10	43	39	4	0.000*
≥10	36	2	34	
Platelet count,/mm3				
<214	43	18	25	0.067
≥214	37	24	13	
Leukocytosis				
Yes	26	9	17	0.033*
No	54	33	21	
Anemia				
Yes	42	25	17	0.262
No	38	17	21	
Lymphocyte counts,/mm3				
<1673	43	31	12	0.000*
≥1673	37	11	26	
Hypoalbuminemia				
Yes	28	26	2	0.001*
No	52	16	36	
PNI				
<44	39	1	38	0.000*
≥44	41	41	0	
Survival status				
Dead	41	3	38	0.000*
Alive	39	39	0	

CRP: C-reactive protein; PNI: prognostic nutritional index; * *P*<0.05 is considered significant.

**Figure 2:**
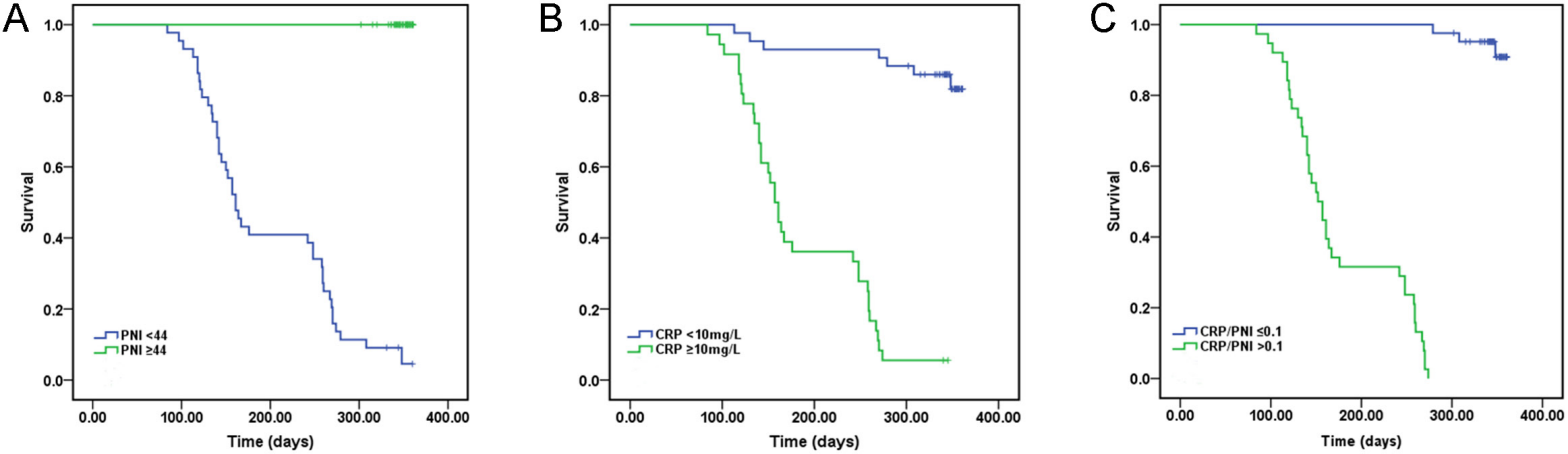
Kaplan-Meier survival curves stratifed by CPR, PNI and CRP/PNI ratio **(A and B)** Patients with elevated CRP (*P* <0.001) or decreased PNI (*P* <0.001) were associated with decreased 1-year survival, respectively. **(C)** Patients with CRP/PNI ratio ≤0.10 had a better 1-year survival than patients with CRP/PNI ratio >0.10 (*P* <0.001). CRP: C-reactive protein; PNI: prognostic nutritional index.

Concerning survival, only 3 of 42 (7.1 %) patients in the CRP/PNI ratio ≤0.10 group died versus 38 of 38 (100.0 %) in the CRP/PNI ratio >0.10 group (Table 2). The survival was significantly influenced by only CRP (*P* <0.001), lymphocyte counts (*P* =0.013), hypoalbuminemia (*P* =0.002), PNI (*P* <0.001) and CRP/PNI ratio (*P* <0.001), while all variables were compared separately to survival status (Table 2). In univariate analysis, the Kaplan–Meier survival curves showed to no significant relationship between platelet count, leukocytosis, anemia and survival. The Kaplan–Meier survival curves of low CRP/PNI ratio versus high CRP/PNI ratio showed a highly significant separation (*P* <0.001, Figure 2C). Furthermore, patients with high CRP (*P* <0.001, Figure 2A) or low PNI (*P* <0.001, Figure 2B) were also related to the poor 1-year survival, respectively. When a multivariate Cox proportional hazard model was constructed (including age, duration of surgery, weight, length of stay, sex, CRP, PNI, platelet count, leukocytosis, anemia, lymphocyte counts, hypoalbuminemia and CRP/PNI ratio), the CRP/PNI ratio was the strongest independent predictor of survival (Table 3).

**Table 2:** Survival status and clinicopathological parameters in 80 specimens

	Case(n)	Survival status		*p* Value
		Dead=41	Alive=39	
Age (years)	86±5	87±5	85±4	0.059
Men	35	19	16	0.142
Weight (kg)	62 (49–78)	60 (49–72)	63 (50–78)	0.699
Length of stay (day)	7 (5–14)	8 (6–14)	7 (5–12)	0.734
Duration of surgery (min)	140 (111–177)	146 (112–177)	139 (111–168)	0.209
Comorbidity				
Diabetes mellitus	34	20	14	0.550
Cardiovascular disease	24	13	11	0.673
Hypertension	63	33	30	0.897
Renal disefficiency	21	11	10	0.803
CRP, mg/L				
<10	43	7	36	0.000*
≥10	36	34	2	
Platelet count,/mm3				
<214	43	26	17	0.116
≥214	37	15	22	
Leukocytosis				
Yes	26	17	9	0.098
No	54	24	30	
Anemia				
Yes	42	18	24	0.125
No	38	23	15	
Lymphocyte counts,/mm3				
<1673	43	13	30	0.013*
≥1673	37	28	9	
Hypoalbuminemia				
Yes	28	26	2	0.002*
No	52	15	37	
PNI				
<44	39	39	0	0.000*
≥44	42	2	30	
CRP/PNI				
≤ 0.10	42	3	39	0.000*
> 0.10	38	38	0	

CRP: C-reactive protein; PNI: prognostic nutritional index; * *P*<0.05 is considered significant.

**Table 3:** Contribution of various potential prognostic factors to survival by Cox regression analysis in 80 specimens

	Hazard ratio	95 % CI	*P*
Age(years)	1.5	0.775∼2.430	0.158
Duration of surgery (min)	0.7	0.341∼3.776	0.342
Weight (kg)	1.8	0.656∼3.167	0.782
Length of stay (day)	0.2	0.096∼2.034	0.053
Sex	1.9	0.127∼3.907	0.102
CRP, mg/L	3.3	1.337∼8.737	0.054
PNI	0.2	0.028∼0.650	0.067
Platelet count,/mm3	2.0	0.672∼6.101	0.210
Leukocytosis	0.9	0.313∼2.860	0.922
Anemia	1. 3	0.432∼3.811	0.653
Lymphocyte counts,/mm3	2.1	1.093∼6.107	0.059
Hypoalbuminemia	1.4	0.897∼3.357	0.072
CRP/PNI	8.1	1.806∼36.216	0.006*

CRP: C-reactive protein; PNI: prognostic nutritional index; CI: confidence interval; statistical analyses were performed by the log-rank test; * *P*<0.05 is considered significant.

**Figure 3:**
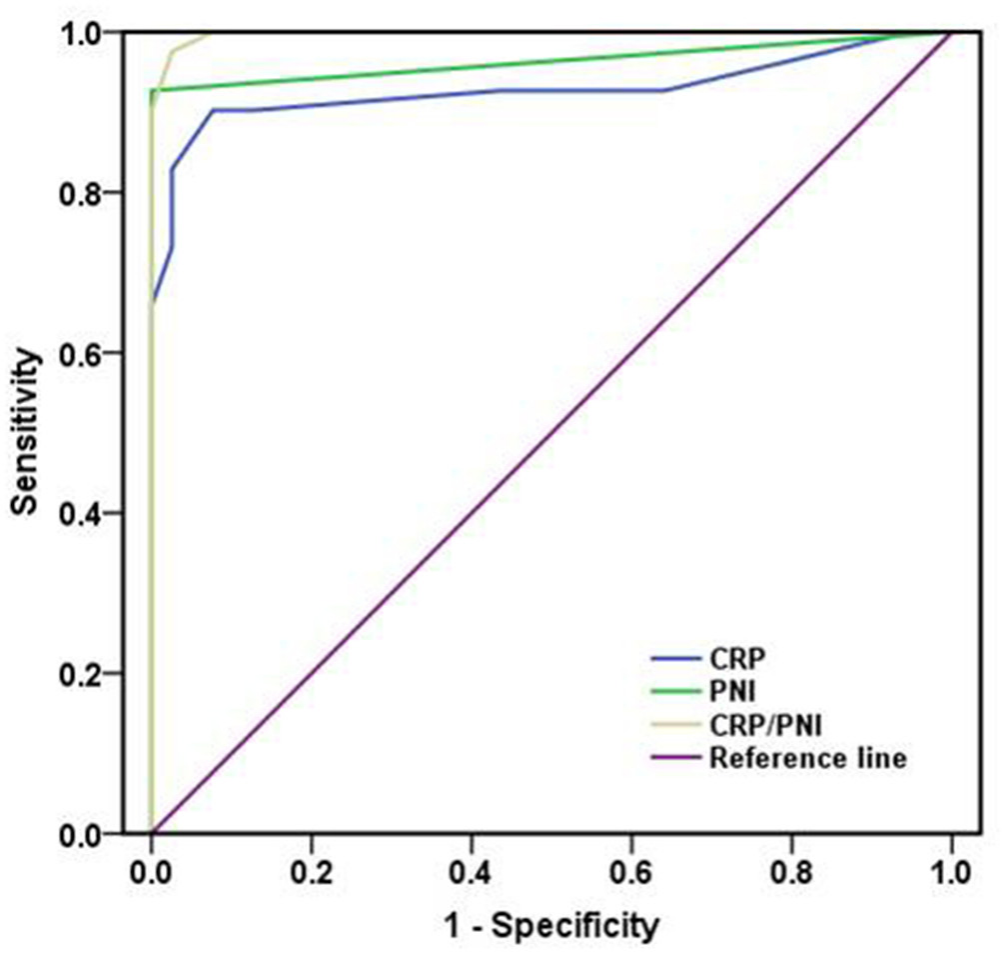
Comparison of the AUC for ROC curves The AUC of the CRP/PNI ratio was higher than other CRP or PNI, indicating that the CRP/PNI ratio was superior to the CRP or PNI for prognosis. CRP: C-reactive protein; PNI: prognostic nutritional index; AUC: area under curve; ROC: receiver operating characteristics.

The areas under the curve (AUC) was 0.998 for CRP/PNI ratio (95% CI: 0.000-1.000, *P* <0.001), 0.928 (95% CI: 0.861-0.990, *P* <0.001) for CRP and 0.953 (95% CI: 0.002-0.996, *P* <0.001) for PNI. Our results indicated that the CRP/PNI ratio was superior to the CRP or PNI (Figure 3) for the older patients after hip fracture surgery.

## DISCUSSION

Because of the poor prognosis of acute surgery in elderly patients, biomarkers in clinical management of these patients are important. In our study, CRP/PNI ratio was used as an independent prognostic factor for elderly patients after orthopedic surgery. And this is the first study which examined the prognostic value of CRP/PNI ratio for elderly patients after orthopedic surgery.

Infection or traumas has a strong relationship with inflammation. Previous data have shown that CRP is a sensitive and non-specific inflammatory marker of human infection or traumas [15]. CRP is proved to be very useful in diagnosis and functioned as a monitor of infections in orthopaedics [13]. A retrospective analysis conducted by Kim et al. [15] revealed that preoperative CRP was an independent risk factor for 1-year mortality after hip fracture surgery in the elderly. In our current research, these patients with low CRP levels (≤10.0 mg/L) had better survival than those with CRP >10.0 mg/L (83.7% vs. 5.6%, *P* <0.001). Regrettably, in the multivariate analyses, there was no evidence to prove that CRP was an independent prognostic factor (*P* =0.054).

The PNI was designed to assess the immunologic and nutritional aspects of surgical patients [16], and calculated by the serum albumin concentration and the lymphocyte count in the peripheral blood [17]. Hypoalbuminemia served as a potential preoperative predictor of outcomes [18]. Lu et al. showed that a lower serum albumin level and total lymphocyte count were important risk factors to predict the one-year mortality of elderly patients with fracture [19]. However, few studies focused on PNI in elderly patients after hip fracture surgery. In our data, PNI was not an independent prognostic factor (*P* =0.067).

As CRP and PNI are affected by various conditions, the CRP/PNI ratio could therefore reduce the potential bias. The prognostic value of CRP/PNI ratio is better than CRP or PNI in elderly patients after hip fracture surgery. In this study, the CRP/PNI ratio has been investigated to assess the outcomes of the elderly patients after hip fracture surgery. Patients with low CRP/PNI ratio had a longer median survival time than those with high CRP/PNI ratio (88.6% vs. 0%, *P* <0.001). CRP/PNI ratio has been demonstrated as an important predictive factor of 1-year survival by multivariate analyses (*P*=0.006). The CRP/PNI ratio had not been investigated before in hip fracture surgery patients before, especially in elderly patients. This is the first time to elucidate that CRP/PNI ratio is a predictor of hip fracture surgery in elderly patients.

In our Cox regression model, multivariate analyses revealed that the CRP/PNI ratio was an independent prognostic factor. Our data showed that the AUC was higher in CRP/PNI ratio than CRP (0.928) or PNI (0.963) by ROC analyses. High levels of CRP/PNI ratio could help us to avoid adverse consequences. Patients will benefit from the CRP/PNI ratio, especially in the elderly patients.

Nevertheless, several limitations should be paid attention in this study. Firstly, this study is a retrospective and single-institution study. Secondly, the number of patients was not adequate (n =80). Thence, larger prospective studies need to be carried out to confirm these preliminary results.

In summary, current data indicate that CRP/PNI ratio is correlated with 1-year survival in elderly patients after hip fracture surgery. Based on these results, we believe that CRP/PNI ratio is a novel and useful predictive factor in elderly patients after hip fracture surgery.

## MATERIALS AND METHODS

### Patients

The data of 80 elderly patients after hip fracture surgery in Shanghai Pudong Hospital between 2015 and 2016 were collected in our study. The 70 years of age or older patients were included, and the patients were excluded if CRP, platelet count, leukocyte count, lymphocyte counts, hemoglobin and albumin measurements were lacking. Ethical approval was obtained for the study protocols and informed consents were obtained from each patient.

### Data collection

We collected clinical data including age, sex, weight, length of stay, duration of surgery, comorbidity, biological data and survival time. The patients had been tested for serum CRP, platelet count, leukocyte count, lymphocyte counts, hemoglobin and albumin before surgery. At the same time, we carefully reviewed the associated comorbidities of these patients. The biological data were measured by automatic laser nephelometry (BN 100 analyzer, Germany). The normal values of CRP, platelet count, leukocyte count, lymphocyte counts, hemoglobin and albumin were 0–10 mg/L, 125–350 ×10^9^/L, 3.5–9.5×10^9^/L, 1.1–3.2 ×10^9^/L, 110–175 ×10^9^/L, 35–50 g/L, respectively.

### Follow-up and endpoint

The mean follow-up period for these patients was 8.7 months (range: 3–12 months). All patients were followed up by phone monthly. The endpoint was mortality within one year.

### Statistical analysis

All statistical analyses were used the SPSS version 22.0 statistical software. The association between CRP and PNI were studied using the Spearman rank correlation test Survival analysis. For analysis of survival data, Kaplan-Meier curves were constructed, and the log-rank test was used for analysis. Univariate and multivariate analyses were performed using Cox’s proportional hazards model. Differenceswere considered statistically significant when P value was less than 0.05.
